# The out-of-pocket burden of chronic diseases: the cases of Belgian, Czech and German older adults

**DOI:** 10.1186/s12913-021-06259-w

**Published:** 2021-03-17

**Authors:** Veronika Kočiš Krůtilová, Lewe Bahnsen, Diana De Graeve

**Affiliations:** 1 Faculty of Business and Economics, Mendel University in Brno, Zemedelska 1, 613 00 Brno, Czech Republic; 2 Institute for Public Finance and Social Policy, Albert-Ludwigs-University of Freiburg, P.O. Box, 79085 Freiburg, Germany; 3 Faculty of Business and Economics, University of Antwerp, Prinsstraat 13, 2000 Antwerp, Belgium

**Keywords:** Out-of-pocket burden, Chronic diseases, Older adults, Belgium, Czech Republic, Germany

## Abstract

**Background:**

Out-of-pocket payments have a diverse impact on the burden of those with a higher morbidity or the chronically ill. As the prevalence of chronic diseases increases with age, older adults are a vulnerable group. The paper aims to evaluate the impact of chronic diseases on the out-of-pocket payments burden of the 50+ populations in Belgium, the Czech Republic and Germany.

**Methods:**

Data from the sixth wave of the Survey of Health, Ageing and Retirement in Europe is used. A two-part model with a logit model in the first part and a generalised linear model in the second part is applied.

**Results:**

The diseases increasing the burden in the observed countries are heart attacks, high blood pressure, cancer, emotional disorders, rheumatoid arthritis and osteoarthritis. Reflecting country differences Parkinson’s disease and its drug burden is relevant in Belgium, the drugs burden related to heart attack and outpatient care burden to chronic kidney disease in the Czech Republic and the outpatient care burden of cancer and chronic lung disease in Germany. In addition, we confirm the regressive character of out-of-pocket payments.

**Conclusions:**

We conclude that the burden is not equitably distributed among older adults with chronic diseases. Identification of chronic diseases with a high burden can serve as a supplementary protective feature.

**Supplementary Information:**

The online version contains supplementary material available at 10.1186/s12913-021-06259-w.

## Background

Chronic diseases are estimated to cause nearly 90% of all deaths and count for 87% of all lost healthy life years in the European Union (EU) [1]. Chronic diseases impose a considerable burden on individuals and households and have not only health-related and social implications but also economic implications [2, 3]. Economic implications mostly arise from the fact that even if health care access is mostly universal, and coverage is extensive in Europe, some health care goods and services must be paid out-of-pocket. Due to legal regulations, out-of-pocket payments (OOPP) for health care goods and services remain and appear either in the form of cost sharing or direct payments. Those individuals who exhibit a higher morbidity are thus more likely to be confronted with OOPP. Furthermore, if no legal exemptions from OOPP are applied, the chronically ill in particular face a higher financial burden of their budgets due to permanent and increased health care consumption [4]. In addition, the presence of chronic diseases not only influences an individual’s labour market participation and productivity resulting in lower earnings but could also lead to disability, early retirement or other types of dependence on social security systems [2, 5]. These factors further constrain household budgets and significantly increase the financial burden for those with chronic conditions compared to the general population [6].

As the prevalence of chronic diseases increases with age [7] and increasing age is an important predictor of multimorbidity [8, 9], the burden from chronic diseases is especially high among older adults [6]. The combination of economic inactivity, resulting in lower disposable income and increased morbidity, leads to a higher share of their budget being spent on health-related OOPP [10, 11]. As a direct consequence, older adults are the most vulnerable group affected by the OOPP burden [12–14] and forgone health care [15, 16].

Our paper contributes to the discussion about the OOPP burden of older adults and extends it by the dimension of health-related aspects. Based on the fact that the relationship between chronic diseases and OOPP has been shown [9, 10, 19] the objective of the paper is to evaluate the impact of specific chronic diseases on the OOPP burden of older adults. The OOPP burden is defined as a share of net equivalised income spent on OOPP. To provide a comprehensive understanding of this issue in complex health care systems we analyse the situation in Belgium, the Czech Republic and Germany where despite well-developed health care systems with extensive packages of health care services covered and various protective measures against high OOPP implemented, older people still report difficulties to afford payments for health care [31, 47, 48]. We aim to answer the following research questions: Does the OOPP burden of older adults differ according to chronic diseases they have? Does the impact of chronic diseases on the OOPP burden differ between certain types of OOPP -- namely drug, outpatient and inpatient care payments? The identification of these points is crucial for policy makers in order to understand the relevance of chronic diseases for cost sharing policies and to target more efficiently protection of the most vulnerable.

The OOPP burden for older adults including the aspect of various chronic diseases has already been studied for the USA [9, 17, 18] and Australia [19]. However, to the best of our knowledge, studies of the burden of older adults and the association with particular chronic health conditions are rare in Europe. We are only aware of the study by Arsenijevic et al. [10] which includes some European countries, however, they explore a different range of OOPP (including home/nursing care) and for a limited set of chronic diseases. The concept of their study was also different because they focused only on catastrophic payments (OOPP exceeding 10% of income). We believe that our study provides important insights into the burden of OOPP of older adults and associated factors, especially chronic diseases.

The paper is organised as follows: firstly, health care systems in Belgium, the Czech Republic and Germany and their coverage and OOPP are briefly introduced; secondly, data and methods used are described; in the third part we present our results which are further discussed in the following part of the paper; and finally we summarise our main findings.

### Health care coverage and out-of-pocket payments

Belgium, the Czech Republic and Germany have well-developed social health insurance systems covering an extensive package of services – including inpatient and outpatient care and drugs [20–22]. Uninsured services are mainly restricted to non-essential drugs such as drugs for diarrhoea or stomach pain or homeopathic drugs, and treatment or surgery at the patient’s request (e.g. plastic surgery or cosmetic interventions). Social insurance is compulsory, except in Germany where the self-employed and employees above an income threshold are free to opt for private health insurance instead – which is effectively done by about 10% of the population.^1^ Supplementary insurance is a common part of the health care system in Belgium and Germany. In the Czech Republic, there is practically no supplementary insurance and voluntary insurance plays a marginal role (mostly used for travel health insurance, cosmetic surgery). The level of OOPP, expressed as a share of total health expenditure, differs from 13% in Germany to 15% in the Czech Republic and 18% in Belgium [23].

OOPP appear as co-payments, co-insurance and direct payments. All three countries secure a certain financial protection from high OOPP and exemptions from payments are applied for specific vulnerable groups (partial or full). OOPP are briefly described for each country and an overview of OOPP settings is presented in Table 1.

**Table 1:** Overview of OOPP settings and protection measures

Country	Type of Care	Co-payment	Co-insurance	Supplementary payments	Uninsured services	Protection measures
**Belgium**	Outpatient	NA	GP and specialist consultations, GP home visits, physiotherapy, speech therapy, podology, etc.	Non-convention healthcare providers	Individual health services	Household level annual maximum for co-payments and co-insurance from 450 to 1800 EUR according to income. A maximum for the chronically ill reduced by 100 EUR. Lower co-payments and co-insurance for low-income households.
	Drugs	NA	Various % according to drug category	NA	Non-essential drugs (e.g. diarrhoea, stomach pain)	
	Inpatient	Flat rate charge per day for inpatient stay, drugs; per stay for tests, radiology	A	A (e.g. one-person rooms)	Individual health services	
**Czech Republic**	Outpatient	Emergency visit fee 3.5 EUR; flat rate charge for therapeutic aids	NA	NA	Individual health services	Exemptions from emergency visit fee for defined vulnerable groups
	Drugs	Flat rate charge per package	NA	NA	Non-essential drugs (e.g. diarrhoea, cold, pain); contraception	Individual annual maximum for drug co-payments: basic (200 EUR); lowered for younger 18 and older 65 (100 EUR)
	Inpatient	NA	NA	Above standard services (one-person rooms)	Individual health services	NA
**Germany**	Outpatient	Physiotherapy 10 EUR per day	10% (min. 5 EUR and max. 10 EUR) for transport services, therapeutic aids	NA	Individual health services	Individual annual maximum for co-payments: 2% of household gross income; 1% for chronically ill
	Drugs	NA	10% for prescription drugs (min. 5 EUR and max. 10 EUR); 10% for herbal remedies	NA	Non-essential drugs (e.g. diarrhoea, stomach pain)	
	Inpatient	Flat rate charge 10 EUR per day for a maximum of 28 day per year	NA	Above standard services (one-person rooms)	Individual health services	

*A* applicable, *GP* general practitioner, *NA* not applicable

#### Belgium

The share of co-payments in total OOPP amounts to about 15 to 17% [22] with relatively large differences according to the specific service and circumstances. For example, beneficiaries pay about €4 to €6 and €13 to €14 for a general practitioner (GP) consultation and home visit respectively and €7 to €12 for a specialist consultation. There is a mix of fixed and variable direct payments for hospitalisation. For reimbursed drugs, co-insurance varies between 0 and 80% of the price according to the type of drug.

Supplementary payments (where a physician/health care facility is allowed to charge a price that is higher than the conventional/agreed tariff) are quite common in Belgium for outpatient care and especially for inpatient care in one-person bedrooms. Additional payments in one-person bedrooms can be quite large – up to €1237 on average in 2015 [24].

Regarding financial protection, an annual limit of total OOPP, excluding supplementary payments and uninsured services, is in place at the household level. The maximum limit varies between €450 and €1800 according to income (e.g. a net taxable household income up to €17,780 is considered for a limit of €450; an income above €46,042 for a limit of €1800 (figures for 2015)). Low-income households pay lower co-payments and co-insurance and are better protected against supplementary payments. Furthermore, people with chronic illness (defined as having more than €300 health insurance expenditures during eight consecutive quarters constituting two calendar years) are entitled to a third-party payment scheme for physicians and dentists and a reduction of €100 of their maximum payment ceiling. Other benefits are conditional, e.g. individuals can also receive an allowance varying between €300 and €600 if they have paid at least €450 co-payments over two consecutive years and were hospitalised more than six times or more than 120 days in a year, received specific nursing care, etc.

#### Czech Republic

More than half of all OOPP is paid for drugs either in the form of direct payments for over-the-counter (OTC) pharmaceuticals or co-payments for prescribed drugs. Co-payments for prescribed drugs are set as flat charges based on a drug category reflecting a difference between a price of the drug in pharmacies and a set reimbursement from health insurance. In outpatient care, OOPP are applied for medical aids (mostly co-payments) and an emergency unit visit fee of €3.5. It is claimed that there might be some forms of informal payments for inpatient care, however, there are no estimates of the real magnitude [20]. Typical direct payments in inpatient care are payments for one-person bedrooms.

Regarding financial protection, an individual annual maximum (only) for drug co-payments of €200 is in place. The annual limit is reduced to €100 for individuals aged 18 years or younger and 65 years or older (the state in 2015). However, a full co-payment for medication is not always included in the limit. The amount of co-payment included within the limit corresponds to the co-payment of the cheapest available drug with the same treatment substance and means of application. In addition, provided that the given drug is in the group of therapeutically interchangeable drugs (the same drug fully reimbursed from health insurance is available) the co-payment is not included in the protective limit at all. Specific vulnerable groups - such as individuals below subsistence income level, children in foster homes or orphanages, or older adults in nursing homes with limited income - are fully exempted only from the emergency unit visit fee. There are no other special exemptions in the health care system.

#### Germany

Statutorily insured persons are obliged to pay co-payments and co-insurance for health services and drugs reimbursed from statutory health insurance. In principle, for drugs, therapeutic aids and travel expenses insured persons pay 10% of the costs, but at least €5 and no more than €10. However, co-payments will never exceed the total cost. There are special regulations for herbal remedies, ambulant home care and physiotherapy. For remedies, it is a co-insurance of 10% of the selling price. Physiotherapy (either outpatient or inpatient) requires €10 per day. In inpatient care, people have to pay €10 per day for a maximum of 28 days. Regarding financial protection, each insured person must pay OOPP only up to his or her individual limit per calendar year. This burden limit accounts for 2% of the family gross income. A lower burden limit of 1% applies for the chronically ill. Further, for insured persons receiving social assistance the burden limit relates to the received social allowance. If these maximum limits are reached, people are exempted from further co-payments and co-insurance within the respective year. However, the application of the burden limit is not automatic and is based on request. A compact overview of the German OOPP regulations can be found elsewhere [21, 25].

## Methods

### Data source and variables used

For our analysis we use the sixth wave of the Survey of Health, Ageing and Retirement in Europe (SHARE) conducted in 2015 [26]. SHARE is a multidisciplinary panel database of cross-national micro data containing detailed information from the areas of economics, health, and psychology as well as social and family networks [27]. In that regard, it covers the population aged 50 years and older of most European countries and Israel. Malter and Börsch-Supan [28] document questionnaire innovations, methodological improvements, new procedures and other important changes introduced during the sixth wave. Our target population are the non-institutionalised individuals aged 50 and older from Belgium, the Czech Republic and Germany.

#### Dependent variable(s)

In the used data, OOPP are defined as payments made directly to health care providers without any reimbursement from a third party and co-payments (co-insurance) for services partly paid by a third party. We adopt a relative definition of the OOPP burden in our study which stems from the WHO’s concept of fairness in financial contributions for health care and households’ capacity to pay for health care [29, 30]. We refer to a household’s net income as the capacity to pay, which is commonly used for studies focusing on the OOPP burden [10, 11, 19, 31, 32]. The dependent variable, the OOPP burden, is defined as the share of OOPP in equivalised net income. Due to economies of scale in consumption using equalised income allows us to account for differences in household size. We construct the equivalised income by applying the square root scale as a more recent method used by OECD [33]. In addition to looking at the total OOPP burden, we use the burden of the respective OOPP types: payments for drugs (prescribed drugs and OTC pharmaceuticals), outpatient care (contacts with medical doctors, qualified nurses, therapists, emergency rooms, outpatient clinic visits, including expenses for diagnostic examinations, excluding dentist visits) and inpatient care (stays in hospitals and other health care facilities) as dependent variables to gain further insights. Table 2 shows the distribution of OOPP burdens in respective countries for respective OOPP types.

**Table 2:** Distribution of OOPP burden in 2015 (in Euro/in % of equivalent income) (wave 6)

Country	Total OOPP	Drugs	Outpatient care	Inpatient care
Belgium	440.54/2.19 (536.34/3.13)	235.35/1.19 (313.30/1.87)	166.15/0.78 (310.86/1.63)	39.03/0.20 (209.90/1.25)
Czech Republic	113.51/1.76 (132.63/2.25)	72.14/1.14 (81.84/1.46)	37.17/0.55 (93.76/1.52)	4.19/0.06 (33.13/0.51)
Germany	299.34/1.44 (521.20/2.70)	92.48/0.48 (121.74/0.72)	183.83/0.83 (476.84/2.40)	23.54/0.12 (94.23/0.55)

Note: average burden expressed in the absolute term in Euro/average burden expressed in the relative term in % of equivalent income; standard deviation in parentheses

#### Independent variables

As OOPP appear at the point of use, the investigation of the burden closely relates to health care consumption and its patterns. Therefore, the primary set of independent variables for our analysis rests on the “Andersen model” for the utilisation of health care services [34]. This approach classifies three categories of individual determinants influencing health care utilisation, namely predisposing, need and enabling factors. Predisposing factors exist prior to the presence of a certain illness and include the consumer’s predictable characteristics such as age, gender, marital status, previous health behaviour, race or ethnicity, family size and its composition, religion, and region of the country and residence. These factors are extended by the beliefs health care consumers have (values concerning health, knowledge about disease and treatment, and attitudes towards health care services). Need factors represent the most urgent reason to utilise health care as they relate to the onset of illness. They distinguish subjective need (self-perceived health status, symptoms of illness and disability) and objectively recognised need (physician-rated urgency of present condition, stated diagnoses, symptoms, etc.). Enabling factors ensure the possibility of access to health care. Factors such as several sources of income, education, insurance coverage, the regular source of care and the price of health services are considered.

The selected variables for our analyses are further benchmarked with those used in previous studies focusing on the OOPP burden [10, 11, 19, 31, 32, 35]. The final set of selected variables is based on results of multicollinearity and goodness-of-fit tests (see section Methodology).

The need factors are of main importance. We incorporate health-related variables which are of a subjective as well as objective nature. To answer our research questions, we include 15 diagnosed health conditions as dummy variables and a group having other conditions for a complete picture. In our study, we use the term “chronic diseases” for the observed health conditions which are in accordance with perception of chronic diseases in a broader term [36]. Table 3 presents these diseases in a more detail.

**Table 3:** Description of observed chronic diseases

Chronic diseases	
Heart attack	Heart attack including myocardial infarction, coronary thrombosis or any other heart problem including congestive heart failure
High blood pressure	High blood pressure or hypertension
High blood cholesterol	High blood cholesterol
Stroke	Stroke or cerebral vascular disease
Diabetes	Diabetes or high blood sugar
Lung disease	Chronic lung disease such as chronic bronchitis or emphysema
Cancer	Cancer or malignant tumour, including leukaemia or lymphoma, but excluding minor skin cancers
Ulcer	Stomach or duodenal ulcer, peptic ulcer
Parkinson	Parkinson’s disease
Cataracts	Cataracts
Alzheimer	Alzheimer’s disease, dementia, organic brain syndrome, senility or any other serious memory impairment
Emotional disorders	Other affective or emotional disorders, including anxiety, nervous or psychiatric problems
Rheumatoid arthritis	Rheumatoid arthritis
Osteoarthritis	Osteoarthritis, or other rheumatism
Chronic kidney disease	Chronic kidney disease
Other conditions	Other not mentioned chronic conditions and other non-chronic conditions (e.g. fractures)

Note: Descriptions are taken from the SHARE main questionnaire for the sixth wave from the variable PH006_DocCond: Has a doctor ever told you that you had/ Do you currently have any of the conditions on this card? (With this we mean that a doctor has told you that you have this condition, and that you are either currently being treated for or bothered by this condition)

Further, limitations in basic activities (ADL; dressing, eating, walking, hygiene and using the toilet) and in instrumental activities of daily living (IADL; shopping, housekeeping, accounting, food preparation and taking medication) enter the model. Both are dummy variables, taking the value 1 if limitations exist. Finally, we control for predisposing (socio-demographic characteristics) and enabling (economic) factors. Socio-demographic variables observed are gender, age (recoding into three categories: 50 to 64, 65 to 79 and 80+) and marital status (distinguishing four states: living in cohabitation, separated, never married, widowed). The enabling factors included are education (measured according to the International Standard Classification of Education - ISCED-97 but recoded into three categories; having none/primary education, secondary and tertiary education) and income (income distribution expressed as equalised income quartiles). Lastly, we include a variable for supplementary health insurance related to institutional settings of the observed health care systems. Table 4 displays sample characteristics of respective variables for the three countries.

**Table 4:** Sample characteristics (percentage shares)

Variables	Belgium	Czech Republic	Germany
Gender			
• female	54.92	59.42	52.46
• male	45.08	40.58	47.54
Age			
• 50–64	48.17	35.84	45.73
• 65–79	38.45	51.90	44.01
• 80 +	13.38	12.25	10.26
Marital status			
• cohabitation	66.82	63.80	73.98
• seperated	14.09	14.05	9.87
• never married	5.98	2.16	4.92
• widowed	13.12	20.00	11.23
Education			
• none/primary	37.60	38.32	11.54
• secondary	27.36	47.82	57.15
• tertiary	35.03	13.86	31.31
Income			
• 1st quartile	25.00	25.00	25.00
• 2nd quartile	25.00	25.00	25.00
• 3rd quartile	25.00	25.00	25.00
• 4th quartile	25.00	25.00	25.00
Heart attack	9.34	12.65	10.31
High blood pressure	34.01	52.29	42.41
High blood cholesterol	30.36	25.35	18.20
Stroke	3.38	4.23	3.51
Diabetes	11.25	19.85	14.07
Lung disease	7.06	7.60	8.01
Cancer	5.05	4.74	6.10
Ulcer	5.66	3.96	2.07
Parkinson	0.72	1.04	0.86
Cataracts	7.22	10.05	8.75
Alzheimer	1.29	1.95	1.60
Emotional disorders	8.08	5.14	7.71
Rheumatoid arthritis	6.63	10.66	11.95
Osteoarthritis	32.16	28.61	21.61
Chronic kidney disease	1.60	2.50	2.11
Other conditions	16.74	18.60	18.59
ADL	14.64	13.20	10.47
IADL	21.25	20.48	14.00
Supplementary health insurance	81.67	6.07	29.18
N	5566	4726	4308

### Methodology

As the dependent variable belongs to the class of limited dependent variables features such as zero mass, non-negativity, right-skewness and heavy tails make more sophisticated handling necessary.^2^ In order to deal with these issues, the literature provides a variety of different approaches [37, 38]. The most straightforward approach which is well-suited to modelling individual health care consumption and burden and is able to deal with the mentioned features is the two-part model (2 PM) [39–41]. An alternative approach is the sample selection model (SSM) [42]. However, a long debate about how to model health expenditures with zero mass leaves us with the suggestion to compare both methods and the model performance [43]. Following this, we ran our models using both methods, then compared model diagnostics and further checked the condition index and combined it with a z-test. Based on this we choose the 2 PM over the SSM.

The 2 PM assumes a sequential decision and decomposes OOPP made into two random components and models each separately. This leads to predictions for the first part -- the probability of having any OOPP -- and for the second part -- the expected burden of OOPP conditional upon having any OOPP. With these two values, we are able to predict the mean share of OOPP in income, conditional on the included independent variables. The first part -- the probability of observing positive OOPP versus zero expenses -- we estimate by means of a logit model. Conditional on having positive OOPP, we then estimate a generalised linear model (GLM) for the second part. Another common option for the second part is to use OLS on the log-transformed dependent variable. However, GLM has the advantage that it eases the homoscedasticity and normality assumptions and weakens bias due to retransforming to the original scale. GLM makes it necessary to specify on the one hand a family that reflects the mean-variance relationship, and on the other hand a link function which provides the relationship between the linear predictor and the mean of the distribution function. To identify the correct family and link, we apply several tests, namely the modified Park test, the Pregibon link test, the Pearson correlation test and the modified Hosmer-Lemeshow test. Additionally, Akaike and Bayesian information criteria, log-likelihood and deviance values are used. Diagnostics yield a gamma distribution with a power link of 0.2 for Belgium, a Poisson distribution with a power link of 0.5 for the Czech Republic and a gamma distribution with a power link of 0.5 for Germany.

We ran four regression models (a main model for total OOPP burden, models for drugs, outpatient and inpatient care burden) for each country separately with these specifications in order to distinguish country-specific differences within the observed period. We report the results of the 2 PM as average marginal effects (AMEs) which show the incremental effects for the complete 2 PM (logit and GLM combined). The results for the sensitivity analysis of the main model can be found in the supplementary files.

## Results

### Factors affecting the OOPP burden

Presenting results, we first focus on the factors affecting the total OOPP burden (Table 5) and then briefly describe and compare it with the results of the various OOPP types (Table 6). Special attention is paid to the need factors as they are of main interest.

**Table 5:** Average marginal effects (AMEs) of the independent variables on the total OOPP burden

Variables	Total OOPP burden		
	BE	CZ	DE
Male gender^a^	−0.412^***^ (0.0773)	−0.322^***^ (0.0603)	− 0.210^**^ (0.0773)
Age^b^			
• 65–79	0.202^*^ (0.0807)	− 0.113 (0.0675)	0.0679 (0.0778)
• 80+	0.351^**^ (0.134)	−0.122 (0.109)	0.229 (0.143)
Marital status^c^			
• separated	− 0.119 (0.107)	0.0750 (0.104)	− 0.0372 (0.133)
• never married	−0.133 (0.213)	− 0.103 (0.201)	− 0.0241 (0.169)
• widowed	−0.407^***^ (0.100)	−0.112 (0.0842)	− 0.163 (0.110)
Education^d^			
• secondary	0.182 (0.0955)	0.217^***^ (0.0614)	0.272^*^ (0.124)
• tertiary	0.258^**^ (0.0953)	0.513^***^ (0.0908)	0.463^***^ (0.136)
Income^e^			
• 2nd quartile	−1.030^***^ (0.139)	−0.581^***^ (0.106)	− 0.134 (0.134)
• 3rd quartile	−1.623^***^ (0.138)	−0.981^***^ (0.103)	− 0.460^***^ (0.123)
• 4th quartile	−2.241^***^ (0.135)	−1.379^***^ (0.0997)	−1.011^***^ (0.117)
Heart attack	0.611^***^ (0.103)	0.556^***^ (0.0887)	0.365^**^ (0.118)
High blood pressure	0.359^***^ (0.0771)	0.376^***^ (0.0600)	0.336^***^ (0.0822)
High blood cholesterol	0.209^**^	0.0339	−0.104
	(0.0782)	(0.0686)	(0.0882)
Stroke	0.281 (0.187)	0.280 (0.155)	0.328 (0.169)
Diabetes	0.478^***^ (0.106)	0.379^***^ (0.0724)	0.0795 (0.0959)
Lung disease	0.588^***^ (0.126)	0.149 (0.107)	0.586^***^ (0.163)
Cancer	0.619^***^ (0.147)	0.333^**^ (0.127)	0.726^***^ (0.177)
Ulcer	0.226 (0.133)	0.0990 (0.149)	0.0462 (0.189)
Parkinson	1.584^***^ (0.355)	0.524^*^ (0.252)	0.486 (0.341)
Cataracts	0.398^**^ (0.147)	0.254^*^ (0.101)	0.140 (0.140)
Alzheimer	0.132 (0.262)	−0.101 (0.203)	0.271 (0.233)
Emotional disorders	0.708^***^ (0.127)	0.375^**^ (0.128)	0.330^*^ (0.144)
Rheumatoid Arthritis	0.393^**^ (0.141)	0.212^*^ (0.0976)	0.311^**^ (0.110)
Osteoarthritis	0.589^***^ (0.0787)	0.457^***^ (0.0650)	0.276^**^ (0.0914)
Chronic kidney disease	0.273 (0.191)	0.519^**^ (0.193)	0.0891 (0.206)
Other conditions	0.589^***^ (0.0951)	0.387^***^ (0.0718)	0.364^***^ (0.0831)
ADL	0.302^*^ (0.121)	0.370^***^ (0.0983)	0.0610 (0.148)
IADL	0.381^***^ (0.0990)	0.259^**^ (0.0818)	−0.0479 (0.122)
Suppl. health insurance^f^	0.128 (0.0900)	−0.0232 (0.122)	0.146 (0.0798)

Note: standard errors in parentheses; * *p* < 0.05, ** *p* < 0.01, *** *p* < 0.001

Reference categories: ^a^ female gender, ^b^ 50–64, ^c^ cohabitation, ^d^ none/primary, ^e^ 1st quartile, ^f^ no supplementary insurance

**Table 6:** Average marginal effects (AME) of the independent variables on drugs, outpatient and inpatient care burden

Variables	Drugs burden			Outpatient care burden			Inpatient care burden		
	BE	CZ	DE	BE	CZ	DE	BE	CZ	DE
Male gender^a^	−0.186^***^ (0.0417)	− 0.117^***^ (0.0337)	− 0.0245 (0.0188)	− 0.183^***^ (0.0411)	− 0.133^**^ (0.0429)	− 0.184^**^ (0.0661)	− 0.0297 (0.0290)	−0.0436^***^ (0.0121)	0.0306^*^ (0.0152)
Age^b^									
- 65-79	0.162^***^ (0.0431)	0.0177 (0.0363)	0.0222 (0.0187)	0.000619 (0.0444)	−0.119^*^ (0.0479)	0.0276 (0.0661)	0.0779^*^ (0.0318)	0.0223 (0.0129)	0.0135 (0.0151)
- 80+	0.272^***^ (0.0763)	0.0923 (0.0701)	0.0871^*^ (0.0374)	0.0177 (0.0733)	−0.218^**^ (0.0687)	0.108 (0.127)	0.102^*^ (0.0482)	0.0405 (0.0229)	0.0282 (0.0285)
Marital status^c^									
- separated	−0.0118 (0.0576)	−0.0595 (0.0540)	− 0.0350 (0.0301)	− 0.134^*^ (0.0538)	0.0980 (0.0751)	−0.0366 (0.116)	0.00275 (0.0428)	0.0245 (0.0224)	0.0543 (0.0296)
- never married	−0.139 (0.0879)	−0.0109 (0.124)	− 0.0736 (0.0383)	− 0.0867 (0.102)	−0.140 (0.103)	0.0746 (0.159)	0.118 (0.106)	0.0636 (0.0507)	−0.0252 (0.0202)
- widowed	−0.186^**^ (0.0575)	−0.0451 (0.0494)	−0.0264 (0.0322)	− 0.215^***^ (0.0530)	−0.0828 (0.0565)	− 0.182^*^ (0.0906)	− 0.0223 (0.0351)	0.00951 (0.0177)	0.0576^*^ (0.0292)
Education^d^									
- secondary	0.00650 (0.0520)	−0.0146 (0.0356)	0.0443 (0.0304)	0.144^**^ (0.0495)	0.194^***^ (0.0427)	0.236^*^ (0.0991)	0.0411 (0.0380)	0.0290^*^ (0.0119)	0.0310 (0.0190)
- tertiary	0.0260 (0.0523)	0.0892 (0.0525)	0.0545 (0.0332)	0.232^***^ (0.0516)	0.342^***^ (0.0621)	0.456^***^ (0.113)	−0.00461 (0.0333)	0.0955^***^ (0.0290)	−0.00640 (0.0199)
Income^e^									
- 2nd quartile	−0.516^***^ (0.0779)	−0.485^***^ (0.0607)	− 0.145^***^ (0.0351)	− 0.353^***^ (0.0750)	− 0.0938 (0.0758)	− 0.0177 (0.125)	− 0.0492 (0.0506)	0.00579 (0.0180)	0.0119 (0.0259)
- 3rd quartile	−0.836^***^ (0.0742)	− 0.695^***^ (0.0606)	− 0.271^***^ (0.0326)	− 0.558^***^ (0.0775)	−0.265^***^ (0.0697)	− 0.164 (0.115)	−0.165^***^ (0.0473)	− 0.0109 (0.0192)	−0.0479^*^ (0.0231)
- 4th quartile	−1.278^***^ (0.0706)	−0.979^***^ (0.0560)	−0.470^***^ (0.0310)	−0.749^***^ (0.0761)	− 0.362^***^ (0.0676)	− 0.469^***^ (0.108)	− 0.192^***^ (0.0473)	−0.00497 (0.0171)	− 0.0917^***^ (0.0232)
Heart attack	0.419^***^ (0.0647)	0.475^***^ (0.0564)	0.175^***^ (0.0299)	0.139^*^ (0.0544)	0.0405 (0.0640)	0.0437 (0.109)	0.0513 (0.0341)	−0.000490 (0.0138)	0.0835^***^ (0.0204)
High blood pressure	0.290^***^ (0.0428)	0.342^***^ (0.0337)	0.108^***^ (0.0180)	0.0386 (0.0403)	0.0439 (0.0426)	0.214^**^ (0.0712)	0.0262 (0.0288)	0.00485 (0.0129)	0.0331^*^ (0.0140)
High cholesterol	0.163^***^ (0.0426)	0.0514 (0.0389)	0.0340 (0.0215)	0.0187 (0.0419)	−0.0440 (0.0460)	−0.132 (0.0790)	0.0242 (0.0324)	0.0113 (0.0141)	−0.00481 (0.0172)
Stroke	0.149 (0.102)	0.190^*^ (0.0889)	0.167^**^ (0.0514)	−0.0415 (0.0817)	−0.00509 (0.115)	−0.0226 (0.144)	0.136^*^ (0.0586)	0.0425^*^ (0.0215)	0.101^**^ (0.0338)
Diabetes	0.362^***^ (0.0660)	0.327^***^ (0.0441)	0.177^***^ (0.0268)	0.0907 (0.0557)	0.0508 (0.0503)	−0.126 (0.0917)	0.0434 (0.0386)	− 0.0117 (0.0133)	0.0358 (0.0200)
Lung disease	0.470^***^ (0.0762)	0.201^**^ (0.0682)	0.162^***^ (0.0324)	−0.0147 (0.0570)	−0.0555 (0.0683)	0.447^**^ (0.145)	0.0699 (0.0456)	0.0116 (0.0204)	0.0299 (0.0206)
Cancer	0.402^***^ (0.0944)	0.318^***^ (0.0837)	0.180^***^ (0.0448)	0.126 (0.0644)	−0.00712 (0.0731)	0.428^**^ (0.152)	0.0549 (0.0504)	0.0550^*^ (0.0249)	0.0951^***^ (0.0270)
Ulcer	0.242^**^ (0.0749)	0.211^*^ (0.0947)	0.105 (0.0671)	−0.00246 (0.0672)	−0.130 (0.105)	−0.0558 (0.158)	0.00906 (0.0493)	−0.00952 (0.0213)	0.0303 (0.0367)
Parkinson	1.202^***^ (0.251)	0.259 (0.160)	0.262^*^ (0.116)	0.318 (0.199)	0.0971 (0.170)	0.0915 (0.236)	0.0751 (0.101)	0.0952^*^ (0.0422)	0.0451 (0.0465)
Cataracts	0.193^*^ (0.0854)	0.133^*^ (0.0565)	0.0282 (0.0308)	0.144 (0.0782)	0.110 (0.0720)	0.124 (0.122)	0.00390 (0.0437)	−0.0169 (0.0194)	−0.0195 (0.0225)
Alzheimer	0.115 (0.177)	0.0187 (0.133)	0.148 (0.0906)	−0.129 (0.129)	−0.192 (0.142)	0.202 (0.224)	0.0463 (0.0920)	−0.0259 (0.0397)	0.0352 (0.0412)
Emotional disorders	0.465^***^ (0.0704)	0.274^***^ (0.0805)	0.130^***^ (0.0322)	0.116 (0.0645)	0.0612 (0.0957)	0.139 (0.147)	0.0760 (0.0470)	0.0436^*^ (0.0176)	0.0501^*^ (0.0215)
Rheum. Arthritis	0.298^***^ (0.0829)	0.182^***^ (0.0548)	0.137^***^ (0.0320)	0.0801 (0.0722)	0.0187 (0.0733)	0.0933 (0.0953)	−0.0189 (0.0459)	−0.00128 (0.0179)	0.0622^**^ (0.0216)
Osteoarthritis	0.375^***^ (0.0432)	0.320^***^ (0.0376)	0.0897^***^ (0.0216)	0.163^***^ (0.0416)	0.127^**^ (0.0458)	0.171^*^ (0.0786)	0.0350 (0.0302)	0.0238 (0.0139)	0.0389^*^ (0.0163)
Chronic kidney dis.	0.356^**^ (0.127)	0.278^*^ (0.111)	0.0688 (0.0696)	−0.0862 (0.103)	0.253^*^ (0.123)	−0.0239 (0.189)	−0.0276 (0.0598)	0.00802 (0.0258)	−0.0141 (0.0310)
Other conditions	0.395^***^ (0.0529)	0.335^***^ (0.0417)	0.168^***^ (0.0224)	0.146^**^ (0.0487)	0.0313 (0.0489)	0.180^*^ (0.0720)	0.0495 (0.0324)	0.0241 (0.0150)	0.0440^**^ (0.0152)
ADL	0.0828 (0.0681)	0.299^***^ (0.0644)	0.0211 (0.0351)	0.0198 (0.0608)	−0.0445 (0.0668)	−0.00492 (0.132)	0.153^***^ (0.0394)	0.0477^**^ (0.0173)	0.00911 (0.0266)
IADL	0.358^***^ (0.0588)	0.315^***^ (0.0529)	0.0859^**^ (0.0326)	0.0149 (0.0532)	−0.135^*^ (0.0549)	−0.238^*^ (0.119)	0.0173 (0.0335)	0.0357^*^ (0.0162)	0.0565^*^ (0.0229)
Suppl. health insurance.^f^	0.0624 (0.0493)	−0.0497 (0.0611)	0.0297 (0.0187)	0.141^**^ (0.0442)	0.0347 (0.0874)	0.109 (0.0695)	−0.152^***^ (0.0438)	0.00315 (0.0233)	−0.00829 (0.0142)

Note: standard errors in parentheses; * *p* < 0.05, ** *p* < 0.01, *** *p* < 0.001

Reference categories: ^a^ female gender, ^b^ 50–64, ^c^ cohabitation, ^d^ none/primary, ^e^ 1st quartile, ^f^ no supplementary insurance

Regarding the total OOPP burden, six chronic diseases contribute to an increase in the burden in all the observed countries but with a significantly different magnitude, namely heart attack, high blood pressure, cancer, emotional disorders, rheumatoid arthritis and osteoarthritis. Other non-specified conditions also increase the total burden in the observed countries.

In terms of magnitude the largest effects can be observed for Belgium as well as the highest number of chronic diseases significantly associated with the high burden (11 specific health conditions). Considering largest effects, older people diagnosed with Parkinson disease have a by around 1.6 percentage points higher OOPP burden compared to those without this disease (AME = 1.584, *p* < 0.001). Being diagnosed with emotional disorders increases the OOPP burden by around 0.7 percentage points (AME = 0.708, *p* < 0.001), cancer and heart attack by around 0.6 percentage points (AME = 0.619, *p* < 0.001, resp. AME = 0.611, *p* < 0.001). Osteoarthritis and chronic lung disease also have a strong and significant impact on the total OOPP burden.

In the Czech Republic, ten chronic diseases significantly increase the total burden with the largest effects for heart attack (AME = 0.556, *p* < 0.001), Parkinson’s disease (AME = 0.524, *p* < 0.05) and chronic kidney disease (AME = 0.519, *p* < 0.01), followed by a slightly lower contribution to the burden by osteoarthritis (AME = 0.457, *p* < 0.001).

Germany has the lowest number of chronic diseases – seven - having effects on the total burden with the highest magnitude of cancer (AME = 0.726, *p* < 0.001) and chronic lung disease (AME = 0.586, *p* < 0.001). Other health conditions with similar effects are heart attack, high blood pressure, emotional disorders, rheumatoid arthritis and osteoarthritis (listed in descending order according to the AMEs).

The effects of other health variables widely differ between countries. Having limitations in ADL and IADL is a significant predictor for the OOPP burden in Belgium and the Czech Republic. No effect is found for supplementary health insurance.

Regarding control variables with the highest impact, the coefficients for income show the regressive character of the OOPP burden. Compared to the first income quartile, older adults in all other quartiles spend a lower share on OOPP with the lowest share in the highest quartile (except for the second quartile in Germany which is not significantly different from the first one). Comparing the countries, the highest burden is shown for the poorest quartile in Belgium which faces a 2.3 percentage points higher burden than the richest quartile.

### Differences in the burden between certain types of OOPP

When looking at the burden of OOPP for drugs, the results are only partly in accordance with the results of the total OOPP burden. The number of chronic diseases having a significant impact on the drugs burden increases in all the observed countries.

Compared to the model with total OOPP, only Alzheimer’s disease and stroke are not significantly associated with the burden in Belgium. The drugs burden related to Parkinson’s disease is of the highest importance compared to other conditions (a three times higher burden). In the Czech Republic, all the observed chronic diseases defined in the main model except Parkinson’s disease, high blood cholesterol and Alzheimer’s disease are significantly related to the drugs burden with the highest magnitude for heart attack. In Germany, the number of chronic diseases significantly influencing the burden increased to ten health conditions with the highest burden for Parkinson’s disease.

Mentioning control variables with a high impact the consistent negative impact of income is reproduced for the drugs burden in all countries.

Looking at the results for the outpatient care, most of these diseases do not significantly increase the burden. In Belgium, heart attack and osteoarthritis are associated with the outpatient care burden (increasing the burden by around 0.16 to 0.14 percentage point). In the Czech Republic, only osteoarthritis (AME = 0.127, *p* < 0.01) and chronic kidney disease (AME = 0.253, *p* < 0.05) show a significant and positive association. In Germany, relatively high AMEs are found for cancer (AME = 0.428, *p* < 0.01) and chronic lung disease (AME = 0.447, *p* < 0.01) and further with a lower magnitude for high blood pressure and osteoarthritis. The negative effect of income persists for all the quartiles in Belgium, for two richest quartiles in the Czech Republic and the richest quartile in Germany. Additionally, supplementary health insurance in Belgium significantly increases the outpatient care burden.

For the inpatient OOPP burden, only stroke is significantly associated with the inpatient care burden in Belgium. Moreover, supplementary health insurance protects from a high inpatient care burden. In the Czech Republic, being diagnosed with Parkinson’s disease, cancer, stroke and emotional disorders increase the burden for inpatient care even if AME’s are very low (ranging from 0.10 to 0.04). In Germany, seven health diseases are associated with an increasing burden for inpatient care, however, the effects are very small, among them with the highest effects for stroke and cancer. Income is a significant predictor for inpatient care only in Belgium and Germany (with around half smaller effects compared to Belgium) for the two richest quartiles (a regressive character persists).

## Discussion

The OOPP burden varies with chronic diseases and has different magnitudes in the observed countries. Nevertheless, some patterns can be found. The main joint association of the high OOPP burden and chronic diseases is found for heart attack, high blood pressure, cancer, emotional disorders, rheumatoid arthritis and osteoarthritis. Previous research based on SHARE data also found that cardiovascular diseases are a significant predictor for very high (catastrophic) OOPP payments in Belgium and the Czech Republic [10]. Further, the SHARE post-death survey showed that cardiovascular diseases and malignant neoplasms are the most frequent cause of death for older adults [44]. Taking into account the fact that cardiovascular diseases and musculoskeletal disorders are most prevalent in the older population of the observed countries, policy makers should pay special attention to these diseases when they target methods to protect older people from the high OOPP burden.

It is obvious that country differences in the burden of particular diseases are a sign of different health care reimbursements and cost sharing policies. Belgium has a complex cost sharing policy with a high share of supplementary and direct payments which are not included in the protective maximum limit. Only co-payments and co-insurance are included to the limit which may imply that inequalities in the distribution of the OOPP burden for a large number of diseases exist as shown in our study. Even if protective measures are quite extensive in the Belgian health care system they might not be as effective as expected which also a relatively recent study on equity in the Belgian health care system confirms [48]. Based on our findings, the drugs burden should be of particular interest as the majority of chronic conditions increase it with a special focus on drugs related to Parkinson’s disease. Large costs (including high supplementary payments) can be applied to hospitalisation in the Belgian health care system, however, we found that only having stroke is significantly associated with the inpatient care burden. It seems that supplementary insurance works well in this regard and our finding about a negative association with the inpatient care burden supports it.

In Germany, cancer and chronic lung disease were identified as the diseases with the largest magnitude, which is especially due to the outpatient care burden. This might be related to co-payments for transport services and non-physician services which are common. It is worth highlighting that even if Parkinson’s disease is not related to the overall burden, it has the highest magnitude for the drugs burden. Thus, prescription practices in relation to Parkinson’s disease should be examined. A closer look at the inpatient care burden would also be desirable. Even if the burden is low, most diseases were positively associated with it. In particular, co-payments for inpatient stays can further contribute to a high burden for older adults having cancer and heart attack. Thus, it seems that charges per inpatient stay can disproportionally burden patients with some chronic conditions even if a maximum for inpatient stay charges is applied.

In the Czech Republic, mostly OOPP for drugs are common in the health care system which coincide with our findings that the drugs burden should be revised in order to lower the burden for older adults (especially drugs which prevent heart attack). Czech politicians recognise that people should be protected from the high OOPP burden but currently, only one protective mechanism in the form of the OOPP maximum limit for drug co-payments is applied. However, its function can be curbed because not all co-payments are included within the limit. It is applied only to co-payments for prescribed drugs and only about one third of prescribed drugs are eligible for the limit. Thus, older people consuming drugs from the remaining two-thirds of medication items are realistically not protected. Moreover, payments for OTC pharmaceuticals are not monitored, however, they play an important role in total OOPP for drugs based on the official statistics [49]. For individuals aged 65 and more, a lowered maximum for drug co-payments is applied. Nevertheless, age is the only criterion and as the findings about age show, this consideration might be appropriate only to a certain extent. Regarding the outpatient and inpatient care the majority of services are covered which is in accordance with our results. In the outpatient care some direct payments can occur in relation to services of private clinics (e. g. physiotherapists, fees for application of intra-articular injections). OOPP for inpatient care are mostly related only to above-standard room services in the Czech health care system which might explain an association of several diseases with the inpatient care burden but the impact on the overall burden is rather negligible.

We conclude that the impact of specific chronic diseases on the OOPP burden differs between the types of OOPP, and that coping with the drugs burden should have a special priority as the majority of observed diseases are associated with an increasing drugs burden in all three countries. However, other types of OOPP burden can be relevant for some diseases as well. Our findings point out that a focus on the effect of the OOPP types is necessary. Without it, the interpretation of findings for specific chronic conditions could be inaccurate as suggested above.

In addition, we found that the economic situation of older adults is a highly relevant predictor. A regressive pattern of OOPP burden is found in all three countries and is confirmed by findings in previous studies of the older population [19, 32, 45]. This finding shows that income constitutes the best protection against the OOPP burden in all three countries. Income protection is missing in the Czech Republic, thus implementing this mechanism might substantially lower the burden for low-income households. However, setting a correct threshold is essential since our findings showed that even if income protection is implemented and directly related to gross income in Germany, it does not work as protectively as expected. A revision of the threshold seems to be desirable for the poorest, especially. The protection of low-income households is also insufficient in Belgium even if the OOPP maximum is structured according to income. Thus, based on our findings, current income protection implemented in the health care system does not counter regressiveness for older adults.

Finally, our research showed that country specifics play a role. Therefore, we focused only on three countries in more detail in this paper in order to provide a background for evaluating the implications for older adults and suggestions for countries’ health and social policies. An investigation of more EU countries is open for future research.

We leave aside a more detailed discussion about the impact of other predisposing and enabling factors included in the analysis as this was not the main focus of the paper. However, some of the findings might be relevant for future research about various determinants of the OOPP burden.

We are aware of some limitations in our study. The accuracy of findings depends on the available data and its design, and although SHARE is one of the comprehensive sources for investigating older adults currently, we are not able to address accurately several aspects. Firstly, SHARE does not contain more detailed information on particular OOPP types (prescribed versus OTC drugs, types of outpatient care, possible informal payments, etc.). We cannot attribute a particular amount of OOPP to specific chronic diseases as the respondents are not asked about health payments in such detail. In addition, as is mentioned in the introduction, multimorbidity is usual in older age, therefore some diseases appear jointly. In order to reflect this fact, we ran cluster analysis and implemented some interaction terms in the model, but the results did not provide any additional valuable insight. This is supported by another similar study which found that observing individual chronic diseases offers better and more useful information about the OOPP burden than clusters or other groupings of diseases [19]. Secondly, after comparing reported OOPP in SHARE with OOPP reported in household budget surveys it seems that health expenditure in SHARE is underestimated, as also pointed out elsewhere [46]. The fact that reported OOPP are based on the respondent’s recall and thus might be linked with better cognition for some respondents contributes to the limitations of our study. Thirdly, regarding the OOPP burden we are not able to distinguish that a part of OOPP reflects a willingness to purchase either OTC pharmaceuticals or other not covered services such as spa-treatment or preventive physiotherapy (necessary versus unnecessary health care services). Fourthly, we did not address possible unmet need due to financial constraints because only unmet need for physician’s visits could have been observed. Fifthly, we used an umbrella term “chronic diseases” for observed health conditions even if some might question this classification. However, there is no generally recognised classification of chronic diseases and the list of chronic diseases differs depending on the defining body [36]. Nevertheless, all observed diseases are highly prevalent among older groups of population and require ongoing medical attention or limit activities of daily living or both. Further, we acknowledge that the empirical analysis might suffer from the endogeneity of some of our explanatory variables, such as income and education. Lastly, it is obvious from the results that the group of other health conditions (chronic and non-chronic) for which we cannot estimate the effect separately play a role as well.

## Conclusions

Specific chronic diseases increase the overall OOPP burden in the observed countries with a strong association for the drug burden. The diseases increasing the overall burden in Belgium, the Czech Republic and Germany are heart attack, high blood pressure, cancer, emotional disorders, rheumatoid arthritis and osteoarthritis. Reflecting country differences, it is worth paying attention to Parkinson’s disease and its drugs burden in Belgium; to the drugs burden related to heart attack and outpatient care burden of chronic kidney disease in the Czech Republic and outpatient care burden of cancer and chronic lung disease in Germany. It seems that only individuals with Alzheimer’s disease are protected well from any OOPP burden in the observed countries.

The OOPP burden is not equitably distributed among individuals with chronic diseases. Even if some protective mechanisms and exemptions from OOPP are implemented in the observed health care systems, some gaps in the protection are obvious. Disease-based protection is a specific protective feature which has to be applied on a national basis and in accordance with morbidity and cost sharing settings in each country. Conversely, income-based protection seems to be an essential universal instrument if we focus on protecting the most vulnerable. Our study points out that evaluating and monitoring the OOPP burden not only from the socio-economic perspective (as a majority of studies do) but also from a disease aspect should be of particular interest because some of the chronic diseases increase the burden considerably.

## Supplementary Information

**Additional file 1.**

## Data Availability

The data used in the analyses are accessible from the SHARE website http://share-project.org/home0.html.

## Abbreviations

EU: European Union
OOPP: out-of-pocket payments
SHARE: Survey of Health, Ageing and Retirement in Europe
WHO: World Health Organization
ADL: limitations in basic activities of daily living
IADL: limitations in instrumental activities of daily living
EURO-D: EURO depression
SPH: self-perceived health
2 PM: two-part model
SSM: sample selection model
GLM: generalised linear model
AME: average marginal effect

## Footnotes

1 Specific regulations of private health insurance in Germany will be left out of consideration.

2 The share of zeros, indicating having no OOPP, is 4.60% (Belgium), 7.13% (Czech Republic) and 14.93% (Germany). Values for skewness and kurtosis indicate a highly right-skewed distribution with very heavy tails.
