# Supplementary material for: The out-of-pocket burden of chronic diseases: the cases of Belgian, Czech and German older adults
Source: BMC Health Serv Res. 2021 Mar 17;21:239. doi: 10.1186/s12913-021-06259-w (PMC7967967; doi:10.1186/s12913-021-06259-w)
Supplement: Supplementary file 1 — Additional file 1. [file 12913_2021_6259_MOESM1_ESM.docx]

**The Out-Of-Pocket Burden of Chronic Diseases: The Cases of Belgian, Czech and German Older Adults**

| Table S1: Average marginal effects (AMEs) of the independent variables on the total OOPP burden, hierarchical estimation for Belgium |
| --- |
| \| Variables \| Total OOPP burden \| \| \| \| \| --- \| --- \| --- \| --- \| --- \| \| 1^st^ stage \| 2^nd^ stage \| 3^rd^ stage \| Full model \| \| Heart attack \| 0.832^***^ \| 0.821^***^ \| 0.678^***^ \| 0.611^***^ \| \|  \| (0.113) \| (0.117) \| (0.103) \| (0.103) \| \| High blood pressure \| 0.385^***^ \| 0.349^***^ \| 0.383^***^ \| 0.359^***^ \| \|  \| (0.0863) \| (0.0836) \| (0.0778) \| (0.0771) \| \| High blood cholesterol \| 0.254^**^ \| 0.235^**^ \| 0.212^**^ \| 0.209^**^ \| \|  \| (0.0919) \| (0.0881) \| (0.0792) \| (0.0782) \| \| Stroke \| 0.347 \| 0.326 \| 0.360 \| 0.281 \| \|  \| (0.214) \| (0.201) \| (0.188) \| (0.187) \| \| Diabetes \| 0.603^***^ \| 0.601^***^ \| 0.515^***^ \| 0.478^***^ \| \|  \| (0.119) \| (0.117) \| (0.107) \| (0.106) \| \| Lung disease \| 0.818^***^ \| 0.873^***^ \| 0.648^***^ \| 0.588^***^ \| \|  \| (0.146) \| (0.150) \| (0.125) \| (0.126) \| \| Cancer \| 0.785^***^ \| 0.773^***^ \| 0.673^***^ \| 0.619^***^ \| \|  \| (0.184) \| (0.181) \| (0.144) \| (0.147) \| \| Ulcer \| 0.408^*^ \| 0.388^*^ \| 0.252 \| 0.226 \| \|  \| (0.188) \| (0.167) \| (0.131) \| (0.133) \| \| Parkinson \| 1.658^***^ \| 1.611^***^ \| 1.901^***^ \| 1.584^***^ \| \|  \| (0.398) \| (0.363) \| (0.378) \| (0.355) \| \| Cataracts \| 0.753^***^ \| 0.490^**^ \| 0.412^**^ \| 0.398^**^ \| \|  \| (0.193) \| (0.188) \| (0.146) \| (0.147) \| \| Alzheimer \| 0.837^**^ \| 0.463 \| 0.388 \| 0.132 \| \|  \| (0.311) \| (0.329) \| (0.265) \| (0.262) \| \| Emotional disorders \| 0.969^***^ \| 0.936^***^ \| 0.809^***^ \| 0.708^***^ \| \|  \| (0.154) \| (0.155) \| (0.124) \| (0.127) \| \| Rheumatoid Arthritis \| 0.731^***^ \| 0.670^***^ \| 0.484^***^ \| 0.393^**^ \| \|  \| (0.152) \| (0.161) \| (0.137) \| (0.141) \| \| Osteoarthritis \| 0.896^***^ \| 0.713^***^ \| 0.648^***^ \| 0.589^***^ \| \|  \| (0.0912) \| (0.0886) \| (0.0785) \| (0.0787) \| \| Chronic kidney disease \| 0.287 \| 0.210 \| 0.348 \| 0.273 \| \|  \| (0.220) \| (0.218) \| (0.193) \| (0.191) \| \| Other conditions \| 0.663^***^ \| 0.670^***^ \| 0.662^***^ \| 0.589^***^ \| \|  \| (0.106) \| (0.102) \| (0.0959) \| (0.0951) \| \| Male gender^a^ \|  \| -0.509^***^ \| -0.431^***^ \| -0.412^***^ \| \|  \|  \| (0.0855) \| (0.0777) \| (0.0773) \| \| Age^b^ \|  \|  \|  \|  \| \| - 65-79 \|  \| 0.389^***^ \| 0.205^*^ \| 0.202^*^ \| \|  \|  \| (0.0884) \| (0.0813) \| (0.0807) \| \| - 80+ \|  \| 0.948^***^ \| 0.437^**^ \| 0.351^**^ \| \|  \|  \| (0.167) \| (0.133) \| (0.134) \| \| Education^c^ \|  \|  \|  \|  \| \| - secondary \|  \| -0.149 \| 0.167 \| 0.182 \| \|  \|  \| (0.105) \| (0.0968) \| (0.0955) \| \| - tertiary \|  \| -0.370^***^ \| 0.219^*^ \| 0.258^**^ \| \|  \|  \| (0.0991) \| (0.0960) \| (0.0953) \| \| Marital status^d^ \|  \|  \|  \|  \| \| - separated \|  \| 0.329^*^ \| -0.122 \| -0.119 \| \|  \|  \| (0.153) \| (0.108) \| (0.107) \| \| - never married \|  \| 0.0422 \| -0.132 \| -0.133 \| \|  \|  \| (0.217) \| (0.221) \| (0.213) \| \| - widowed \|  \| -0.212 \| -0.364^***^ \| -0.407^***^ \| \|  \|  \| (0.124) \| (0.104) \| (0.100) \| \| Income^e^ \|  \|  \|  \|  \| \| - 2^nd^ quartile \|  \|  \| -1.005^***^ \| -1.030^***^ \| \|  \|  \|  \| (0.140) \| (0.139) \| \| - 3^rd^ quartile \|  \|  \| -1.615^***^ \| -1.623^***^ \| \|  \|  \|  \| (0.137) \| (0.138) \| \| - 4^th^ quartile \|  \|  \| -2.244^***^ \| -2.241^***^ \| \|  \|  \|  \| (0.134) \| (0.135) \| \| ADL \|  \|  \|  \| 0.302^*^ \| \|  \|  \|  \|  \| (0.121) \| \| IADL \|  \|  \|  \| 0.381^***^ \| \|  \|  \|  \|  \| (0.0990) \| \| Suppl. health insurance^f^ \|  \|  \|  \| 0.128 \| \|  \|  \|  \|  \| (0.0900) \| |
| *Note: Dependent variable: total OOPP burden in %; standard errors in parentheses; * p < 0.05, ** p < 0.01, *** p < 0.001.*  *Reference categories: ^a^ female gender, ^b^ 50-64, ^c^ none/primary, ^d^ cohabitation, ^e^ 1^st^ quartile, ^f^ no supplementary insurance.* |

| Table S2: Average marginal effects (AMEs) of the independent variables on the total OOPP burden, hierarchical estimation for the Czech Republic |
| --- |
| \| Variables \| Total OOPP burden \| \| \| \| \| --- \| --- \| --- \| --- \| --- \| \| 1^st^ stage \| 2^nd^ stage \| 3^rd^ stage \| Full model \| \| Heart attack \| 0.621^***^ \| 0.649^***^ \| 0.598^***^ \| 0.556^***^ \| \|  \| (0.0944) \| (0.0935) \| (0.0877) \| (0.0887) \| \| High blood pressure \| 0.364^***^ \| 0.380^***^ \| 0.372^***^ \| 0.376^***^ \| \|  \| (0.0659) \| (0.0646) \| (0.0602) \| (0.0600) \| \| High blood cholesterol \| 0.0762 \| 0.0565 \| 0.0585 \| 0.0339 \| \|  \| (0.0772) \| (0.0744) \| (0.0690) \| (0.0686) \| \| Stroke \| 0.437^**^ \| 0.482^**^ \| 0.412^**^ \| 0.280 \| \|  \| (0.165) \| (0.161) \| (0.149) \| (0.155) \| \| Diabetes \| 0.430^***^ \| 0.457^***^ \| 0.412^***^ \| 0.379^***^ \| \|  \| (0.0792) \| (0.0780) \| (0.0728) \| (0.0724) \| \| Lung disease \| 0.204 \| 0.210 \| 0.202 \| 0.149 \| \|  \| (0.120) \| (0.118) \| (0.109) \| (0.107) \| \| Cancer \| 0.416^**^ \| 0.420^**^ \| 0.380^**^ \| 0.333^**^ \| \|  \| (0.146) \| (0.144) \| (0.131) \| (0.127) \| \| Ulcer \| 0.202 \| 0.222 \| 0.120 \| 0.0990 \| \|  \| (0.162) \| (0.158) \| (0.149) \| (0.149) \| \| Parkinson \| 0.737^**^ \| 0.828^**^ \| 0.786^**^ \| 0.524^*^ \| \|  \| (0.261) \| (0.259) \| (0.252) \| (0.252) \| \| Cataracts \| 0.363^**^ \| 0.289^**^ \| 0.273^**^ \| 0.254^*^ \| \|  \| (0.112) \| (0.109) \| (0.102) \| (0.101) \| \| Alzheimer \| 0.182 \| 0.140 \| 0.119 \| -0.101 \| \|  \| (0.212) \| (0.212) \| (0.205) \| (0.203) \| \| Emotional disorders \| 0.602^***^ \| 0.490^***^ \| 0.476^***^ \| 0.375^**^ \| \|  \| (0.138) \| (0.138) \| (0.125) \| (0.128) \| \| Rheumatoid Arthritis \| 0.316^**^ \| 0.315^**^ \| 0.262^**^ \| 0.212^*^ \| \|  \| (0.103) \| (0.103) \| (0.0956) \| (0.0976) \| \| Osteoarthritis \| 0.596^***^ \| 0.550^***^ \| 0.511^***^ \| 0.457^***^ \| \|  \| (0.0685) \| (0.0673) \| (0.0642) \| (0.0650) \| \| Chronic kidney disease \| 0.508^*^ \| 0.571^**^ \| 0.578^**^ \| 0.519^**^ \| \|  \| (0.201) \| (0.196) \| (0.193) \| (0.193) \| \| Other conditions \| 0.460^***^ \| 0.437^***^ \| 0.434^***^ \| 0.387^***^ \| \|  \| (0.0779) \| (0.0755) \| (0.0709) \| (0.0718) \| \| Male gender^a^ \|  \| -0.412^***^ \| -0.330^***^ \| -0.322^***^ \| \|  \|  \| (0.0649) \| (0.0606) \| (0.0603) \| \| Age^b^ \|  \|  \|  \|  \| \| - 65-79 \|  \| 0.0299 \| -0.0979 \| -0.113 \| \|  \|  \| (0.0695) \| (0.0677) \| (0.0675) \| \| - 80+ \|  \| 0.221 \| -0.0189 \| -0.122 \| \|  \|  \| (0.115) \| (0.108) \| (0.109) \| \| Education^c^ \|  \|  \|  \|  \| \| - secondary \|  \| 0.0814 \| 0.193^**^ \| 0.217^***^ \| \|  \|  \| (0.0661) \| (0.0617) \| (0.0614) \| \| - tertiary \|  \| 0.0979 \| 0.475^***^ \| 0.513^***^ \| \|  \|  \| (0.0888) \| (0.0902) \| (0.0908) \| \| Marital status^d^ \|  \|  \|  \|  \| \| - separated \|  \| 0.351^**^ \| 0.0825 \| 0.0750 \| \|  \|  \| (0.116) \| (0.105) \| (0.104) \| \| - never married \|  \| 0.247 \| -0.0904 \| -0.103 \| \|  \|  \| (0.236) \| (0.202) \| (0.201) \| \| - widowed \|  \| 0.129 \| -0.109 \| -0.112 \| \|  \|  \| (0.0877) \| (0.0842) \| (0.0842) \| \| Income^e^ \|  \|  \|  \|  \| \| - 2^nd^ quartile \|  \|  \| -0.611^***^ \| -0.581^***^ \| \|  \|  \|  \| (0.107) \| (0.106) \| \| - 3^rd^ quartile \|  \|  \| -1.030^***^ \| -0.981^***^ \| \|  \|  \|  \| (0.104) \| (0.103) \| \| - 4^th^ quartile \|  \|  \| -1.422^***^ \| -1.379^***^ \| \|  \|  \|  \| (0.100) \| (0.0997) \| \| ADL \|  \|  \|  \| 0.370^***^ \| \|  \|  \|  \|  \| (0.0983) \| \| IADL \|  \|  \|  \| 0.259^**^ \| \|  \|  \|  \|  \| (0.0818) \| \| Suppl. health insurance^f^ \|  \|  \|  \| -0.0232 \| \|  \|  \|  \|  \| (0.122) \| |
| *Note: Dependent variable: total OOPP burden in %; standard errors in parentheses; * p < 0.05, ** p < 0.01, *** p < 0.001.*  *Reference categories: ^a^ female gender, ^b^ 50-64, ^c^ none/primary, ^d^ cohabitation, ^e^ 1^st^ quartile, ^f^ no supplementary insurance.* |

| Table S3: Average marginal effects (AMEs) of the independent variables on the total OOPP burden, hierarchical estimation for Germany |
| --- |
| \| Variables \| Total OOPP burden \| \| \| \| \| --- \| --- \| --- \| --- \| --- \| \| 1^st^ stage \| 2^nd^ stage \| 3^rd^ stage \| Full model \| \| Heart attack \| 0.413^**^ \| 0.403^***^ \| 0.349^**^ \| 0.365^**^ \| \|  \| (0.128) \| (0.122) \| (0.116) \| (0.118) \| \| High blood pressure \| 0.382^***^ \| 0.382^***^ \| 0.330^***^ \| 0.336^***^ \| \|  \| (0.0867) \| (0.0874) \| (0.0821) \| (0.0822) \| \| High blood cholesterol \| -0.118 \| -0.133 \| -0.105 \| -0.104 \| \|  \| (0.0964) \| (0.0944) \| (0.0873) \| (0.0882) \| \| Stroke \| 0.376 \| 0.383 \| 0.330 \| 0.328 \| \|  \| (0.194) \| (0.200) \| (0.168) \| (0.169) \| \| Diabetes \| 0.120 \| 0.163 \| 0.0680 \| 0.0795 \| \|  \| (0.0984) \| (0.0990) \| (0.0949) \| (0.0959) \| \| Lung disease \| 0.644^***^ \| 0.664^***^ \| 0.572^***^ \| 0.586^***^ \| \|  \| (0.162) \| (0.160) \| (0.160) \| (0.163) \| \| Cancer \| 0.828^***^ \| 0.809^***^ \| 0.714^***^ \| 0.726^***^ \| \|  \| (0.203) \| (0.202) \| (0.176) \| (0.177) \| \| Ulcer \| 0.0476 \| 0.00975 \| 0.0230 \| 0.0462 \| \|  \| (0.209) \| (0.193) \| (0.187) \| (0.189) \| \| Parkinson \| 0.574 \| 0.495 \| 0.494 \| 0.486 \| \|  \| (0.357) \| (0.348) \| (0.336) \| (0.341) \| \| Cataracts \| 0.309^*^ \| 0.255 \| 0.158 \| 0.140 \| \|  \| (0.156) \| (0.162) \| (0.139) \| (0.140) \| \| Alzheimer \| 0.0221 \| -0.00447 \| 0.231 \| 0.271 \| \|  \| (0.222) \| (0.223) \| (0.224) \| (0.233) \| \| Emotional disorders \| 0.386^*^ \| 0.372^*^ \| 0.351^*^ \| 0.330^*^ \| \|  \| (0.167) \| (0.157) \| (0.146) \| (0.144) \| \| Rheumatoid Arthritis \| 0.392^***^ \| 0.388^**^ \| 0.300^**^ \| 0.311^**^ \| \|  \| (0.118) \| (0.121) \| (0.108) \| (0.110) \| \| Osteoarthritis \| 0.350^***^ \| 0.320^**^ \| 0.276^**^ \| 0.276^**^ \| \|  \| (0.101) \| (0.101) \| (0.0910) \| (0.0914) \| \| Chronic kidney disease \| 0.108 \| 0.125 \| 0.0976 \| 0.0891 \| \|  \| (0.213) \| (0.218) \| (0.206) \| (0.206) \| \| Other conditions \| 0.358^***^ \| 0.354^***^ \| 0.361^***^ \| 0.364^***^ \| \|  \| (0.0899) \| (0.0899) \| (0.0828) \| (0.0831) \| \| Male gender^a^ \|  \| -0.202^*^ \| -0.208^**^ \| -0.210^**^ \| \|  \|  \| (0.0831) \| (0.0775) \| (0.0773) \| \| Age^b^ \|  \|  \|  \|  \| \| - 65-79 \|  \| 0.178^*^ \| 0.0668 \| 0.0679 \| \|  \|  \| (0.0861) \| (0.0776) \| (0.0778) \| \| - 80+ \|  \| 0.369^*^ \| 0.214 \| 0.229 \| \|  \|  \| (0.152) \| (0.137) \| (0.143) \| \| Education^c^ \|  \|  \|  \|  \| \| - secondary \|  \| 0.281^*^ \| 0.282^*^ \| 0.272^*^ \| \|  \|  \| (0.131) \| (0.124) \| (0.124) \| \| - tertiary \|  \| 0.335^*^ \| 0.473^***^ \| 0.463^***^ \| \|  \|  \| (0.146) \| (0.136) \| (0.136) \| \| Marital status^d^ \|  \|  \|  \|  \| \| - separated \|  \| 0.0233 \| -0.0310 \| -0.0372 \| \|  \|  \| (0.131) \| (0.138) \| (0.133) \| \| - never married \|  \| 0.170 \| -0.0351 \| -0.0241 \| \|  \|  \| (0.202) \| (0.170) \| (0.169) \| \| - widowed \|  \| -0.105 \| -0.170 \| -0.163 \| \|  \|  \| (0.120) \| (0.108) \| (0.110) \| \| Income^e^ \|  \|  \|  \|  \| \| - 2^nd^ quartile \|  \|  \| -0.117 \| -0.134 \| \|  \|  \|  \| (0.133) \| (0.134) \| \| - 3^rd^ quartile \|  \|  \| -0.439^***^ \| -0.460^***^ \| \|  \|  \|  \| (0.122) \| (0.123) \| \| - 4^th^ quartile \|  \|  \| -0.982^***^ \| -1.011^***^ \| \|  \|  \|  \| (0.116) \| (0.117) \| \| ADL \|  \|  \|  \| 0.0610 \| \|  \|  \|  \|  \| (0.148) \| \| IADL \|  \|  \|  \| -0.0479 \| \|  \|  \|  \|  \| (0.122) \| \| Suppl. health insurance^f^ \|  \|  \|  \| 0.146 \| \|  \|  \|  \|  \| (0.0798) \| |
| *Note: Dependent variable: total OOPP burden in %; standard errors in parentheses; * p < 0.05, ** p < 0.01, *** p < 0.001.*  *Reference categories: ^a^ female gender, ^b^ 50-64, ^c^ none/primary, ^d^ cohabitation, ^e^ 1^st^ quartile, ^f^ no supplementary insurance.* |
